# Influence of Healthy Habits Counseling on Biochemical and Metabolic Parameters in Children and Adolescents with HIV: Longitudinal Study

**DOI:** 10.3390/nu13093237

**Published:** 2021-09-17

**Authors:** Rafaela Catherine da Silva Cunha de Medeiros, Tatiane Andreza Lima da Silva, Anna Luiza Vasconcelos de Oliveira, Paulo Francisco de Almeida-Neto, Jason Azevedo de Medeiros, Alexandre Bulhões-Correia, Francisco Americo Micussi, Marcela Abbott Galvao Ururahy, Breno Guilherme de Araújo Tinoco Cabral, Paulo Moreira Silva Dantas

**Affiliations:** 1 Postgraduate Program in Health Science, Federal University of Rio Grande do Norte, Natal 59078-970, Brazil; tatianeandreza@yahoo.com (T.A.L.d.S.); jason.medeiros1@hotmail.com (J.A.d.M.); brenotcabral@gmail.com (B.G.d.A.T.C.); 2 Department of Physical Education, State University of Rio Grande do Norte, Mossoró 59600-000, Brazil; 3 Department of Nutrition, Federal University of Rio Grande do Norte, Natal 59078-970, Brazil; vasconcelosannaluiza@gmail.com; 4 Department of Physical Education, Federal University of Rio Grande do Norte, Natal 59078-970, Brazil; alexandrebulhoescorreia@gmail.com; 5 Giselda Trigueiro Hospital, Natal 59078-970, Brazil; micussiped@gmail.com; 6 Department of Clinical and Toxicological Analyses, Health Sciences Center, Natal 59012-570, Brazil; marcelaururahy@yahoo.com.br

**Keywords:** nutrition, physical activity, young, HIV

## Abstract

We analyze the influence of dietary counseling and physical activity on biochemical and metabolic parameters in children and adolescents with HIV. A longitudinal experimental study, including three analyses: At the beginning, 4th month, and 8th month. A sample of 18 subjects with HIV of both sexes, mean age 10.4 ± 4.50 years. Usual food intake (24 h recall and food intake marker), level of habitual physical activity, biochemical parameters, resting metabolic rate, as well as body composition (dual-energy X-ray absorptiometry), biological maturation, and anamnesis with clinical data and socioeconomic were evaluated. There was an effect of time on the reduction of blood glucose and triglycerides and the resting metabolic rate. There was a significant increase in fruit consumption throughout the study. The consumption of soft drinks decreased when comparing analysis periods 1 and 2, however, it increased again in analysis period 3. There was no significant effect of time on the set of variables related to a food recall. Counseling healthy habits and regular clinical follow-up were relevant for improving biochemical parameters (glucose, triglyceride, HDL cholesterol), maintaining the resting metabolic rate, increasing fruit consumption, and decreasing the consumption of soft drinks, in part of the time, of children and adolescents with HIV. Finally, we emphasize that counseling positively influenced healthy habits, and these, in turn, improved health-related parameters.

## 1. Introduction

Despite the high efficacy of highly active antiretroviral therapy (HAART), which provides an increase in the expectation and quality of life of the population with HIV, the chronic use of HAART is related to persistent immune activation and chronic inflammation, increasing health risks with a predisposition to cardiovascular or metabolic diseases [1].

Particularly, in children and adolescents, who go through critical stages of development, the short-and long-term impact may be even greater [2] due to the need for lifelong treatment [3]. The use of medication is also important to preserve neurodevelopment functions [4]. However, it promotes lipodystrophy syndrome (LS)*,* characterized by abnormal redistribution of body fat and metabolic changes, such as changes in the glycemic profile and dyslipidemia [5,6], which compromise the growth and development of this population [7].

When it comes to children and adolescents, these disorders must be observed more carefully since some drugs have a cumulative effect, which is an independent risk factor, others have only a transitory effect [6]. Thus, complications can generate acute, serious, and permanent problems, especially in those infected vertically [8].

In addition to the changes promoted by infection and therapies, children and adolescents with HIV (CAH) tend to be in a situation of social vulnerability, as they live in geographic regions with difficult access to services and continuity of treatment, in addition to having a low educational level and living in poverty [9], thus needing a lot of attention for several issues [10]. For positive effects or improvements in the health status of people living with HIV (PLHIV) to happen, health interventions should seek to improve support to face the challenges of socioeconomic inequalities and morbid conditions that result in hospitalization [11].

Increased physical activity (PA) can improve physical fitness and potentially decrease cardiovascular risk [12], thus, together with dietary guidance for CAH, healthier habits or lifestyles can be achieved [13]. Although many of the benefits of nutritional and PA interventions are well-founded in the literature, further studies are needed on nutritional support through food instead of pills, as well as monitoring and warning about the problems that CAH may have with sedentary behavior [1].

Thus, the present study hypothesizes that if CAH adopt healthy eating habits and have an increase or maintenance of the level of physical activity, positive behaviors in biochemical and metabolic parameters can be achieved over time. Given this context, the objective of the present study was to analyze the influence of dietary counseling and physical activity on biochemical and metabolic parameters in children and adolescents with HIV.

## 2. Materials and Methods

### 2.1. Sample

The research has a longitudinal experimental design. The group underwent an intervention with nutritional counseling and PA, being monitored over time (8 months).

The sample consisted of young people with HIV of both sexes with a mean age of 10.4 ± 4.50. To determine the sample number, we used the data available regarding the pediatric population of the state of Rio Grande do Norte/Brazil (RN) [14]. In regards to the age group of the intended sample, we considered the records of young people with HIV living in the state of RN in 2020. Thus, in 2020, 25 young people with HIV who lived in RN were officially registered [14], being our available population. Subsequently following the recommendations of Sounis [15] for limited sampling, we considered a standard error of 5% and a first approximation expression factor, thus, we reached the minimum sample number of 10 subjects for this research.

Only children and adolescents who were regularly monitored at the Specialized HIV/AIDS Care Service (SCS) in Natal, RN were included, and those who were using medications (for diabetes and growth hormone) that could interfere with the results were excluded.

**Figure 1:**
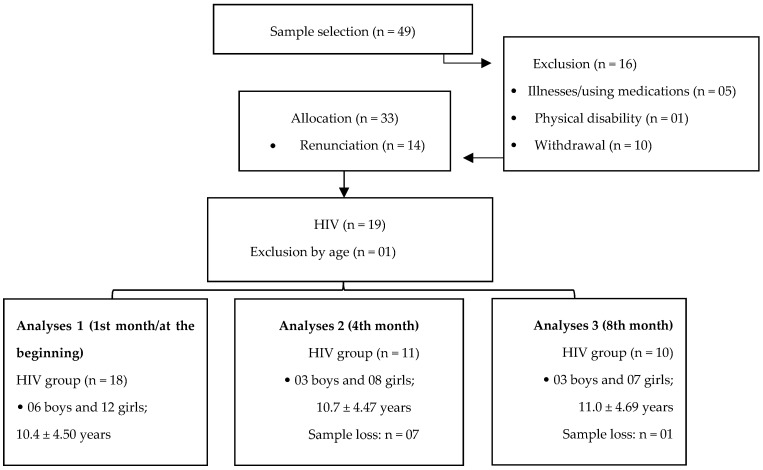
Study flowchart.

Regarding the longitudinal follow-up, the analyses were performed at 4-month intervals. Thus, at the beginning of the study, the sample consisted of 18 subjects (6 male and 12 female). In the second period of analysis, there was a sample loss of 62% concerning the beginning of the study, with only 11 subjects present (3 male and 8 female). In the third stage of analysis, there was a 10% reduction concerning step 2, remaining only ten subjects (3 male and 7 female). Considering that the number of the final sample was still within the parameters of the sample calculation, the analyses of the present study was continued (Figure 1).

### 2.2. Instruments and Procedures

#### 2.2.1. Anamnesis

Through a structured questionnaire, sociodemographic data (age, sex, education, income, number of people in the residence), and data from clinical examinations related to the infection (time of diagnosis, TCD4 + lymphocyte count and most recent viral load, time of use of antiretroviral therapy and combination of drugs).

#### 2.2.2. Biochemical Parameters

The biochemical evaluation was composed of the analysis of the following parameters: Glycemia, lipid profile (total cholesterol, high-density lipoprotein cholesterol (HDL-c), and triglyceride), urea, and creatinine. The value of low-density lipoprotein cholesterol (LDL-c) was calculated using the Friedwald formula.

The determination of biochemical parameters was carried out at the Integrated Laboratory of Toxicological and Clinical Analysis at UFRN, Natal/RN. Such analytes were determined using LABTEST^®^ kits (Lagoa Santa, Minas Gerais, Brazil) and the LABMAX PLENNO equipment (Labtest, Lagoa Santa, Minas Gerais, Brazil), according to the methodology described by the manufacturer.

#### 2.2.3. Resting Metabolic Rate (RMR)

After the volunteer arrived at the laboratory, at 6 a.m., the researchers carried out the assessment of the volunteer’s body mass and height so that these data could be entered into the patient’s registration system for the examination. After such procedure, the volunteer remained at rest in the supine position for 30 min before the evaluation. The evaluation started at 7:30 a.m., under low light conditions, without noise, ambient temperature evaluated at 24 °C, and relative humidity of around 50%.

The oxidation of substrates at rest was evaluated by indirect calorimetry, using the respiratory gas analyzer (Cortex Metalyzer 3B^®^, Biophysik, Leipzig, Germany). During the evaluation, the volunteer remained lying down without moving with a mask fixed on his face and connected to the calorimeter, being instructed to remain awake during the entire evaluation, which lasted 30 min.

From the values of oxygen consumption (VO_2_) and carbon dioxide production (VCO_2_) of the last 25 min of the exam, the respiratory quotient “R” was determined. The gas analyses were used to quantify the RMR.

All were instructed to have 8 h of sleep the night before, too fast for 10 to 12 h, not to practice physical exercises in the 48 h that preceded the tests, and not to consume beverages containing caffeine in the 24 h that preceded the tests. This approach aimed to reduce the influence of the thermal effect of food and PA on resting metabolism.

#### 2.2.4. Usual Food Intake

The analysis of food intake was composed of two surveys: The 24 h recall (24HR) and the food intake marker from the Food and Nutrition Surveillance System (SISVAN) of the Ministry of Health (Brazil) [16]. The 24HR, the method chosen to assess habitual food intake, was answered in two moments, one referring to a day of the week (Monday to Friday) and the other referring to a day of the weekend (Saturday or Sunday).

To perform the 24HR, a photographic record was used with the usual portions of some foods to get as close as possible to the portions consumed. The questions were initially directed to the child or adolescent, with the assistance of the guardian, mainly to describe the method of preparation and portioning. The first 24HR was completed on the days of the laboratory visits, and on a non-consecutive day, contact was made by phone with the participant or guardian to collect the information from the second 24HR. Thus, evaluating a day of the week and a day of the weekend.

To estimate the total energy value (TEV), individual calculations were performed based on each participant’s weight and age [17]. Nutritional adjustments were estimated from the dietary reference intakes (DRI) [18], as well as by individual analysis of each participant.

The SISVAN [16] food consumption marker evaluated the frequency of ingestion of 10 food groups in the last 7 days. Although it is a short questionnaire, it presents groups of foods capable of representing specific nutrient sources, such as fiber, saturated fats, and simple carbohydrates.

The instruments were applied and analyzed by researchers in the field of Nutrition. For the analysis of 24HR, the nutritional software Web Diet was used, and all data referring to food consumption were tabulated in the Excel software. Foods that did not make up the program’s database were inserted based on nutrition labels.

#### 2.2.5. Habitual Physical Activity Level (HPAL)

The instrument used was a questionnaire aimed at estimating the level of the habitual practice of physical activity proposed by Baecke et al. [19]—Baecke Questionnaire of Habitual Physical Activity (BQHPA), reproducible and validated [19] by Guedes et al. [19]. The BQHPA is self-administered and structured by 16 questions, distributed in three distinct sections, which seek to establish estimates regarding a specific dimension of the level of the habitual practice of physical activity with answers indicated or coded on a five-point Lickert scale. The first part of the instrument refers to the practice of physical activity at school, which goes from question one to eight; the second is dedicated to sports activities, physical exercise programs, and active leisure, from question nine to twelve, and the third part offers indications of activities for occupying free time, ranging from question thirteen to sixteen. The questionnaire was applied in the form of an interview with the volunteers, using the parent’s help when necessary.

#### 2.2.6. Chronological Age Analysis

The chronological age in months was determined by the sum of the individual’s months of life, from his date of birth until the date of analysis of the present study [20]. In this way the sum of months of life was divided by 12, resulting in their chronological age in years [20].

#### 2.2.7. Biological Maturation

Skeletal maturation, used as a control variable, was determined by the equation: Bone Age _years)_—Chronological Age _(years)_. Thus, for this procedure, bone age was verified by Equation [21]:

Bone age = −11.620 + 7.004 × (Height _(m)_) + 1.226 × (Dsex) + 0.749 × (Age _(years)_)−0.068 × (Triceps Skinfold _(mm)_) + 0.214 × (Corrected Arm Circumference _(cm)_)−0.588 × (Humerus Diameter _(cm)_) + 0.388 × (Femoral Diameter _(cm)_) (1)

For the male Dsex = 0. For the female Dsex = 1. (m): Meters. (mm): Millimeters. (cm): Centimeters.

To estimate somatic maturation, the peak height velocity (PHV) was used through the following Equation [22]:

Maturity offset in males = −9.236 + [0.0002708 × (Leg length _(cm)_ × Trunk Height _(cm)_)] + [−0.001663 × (Age _(years)_ × Leg length _(cm)_)] + [0.007216 × (Age _(years)_ × Trunk Height _(cm)_)] + [0.02292 × (Weight _(kg)_/Height _(cm)_) × 100] (2)

Maturity offset in females = −9.376 + [0.0001882 × (Leg length _(cm)_ × Trunk Height _(cm)_)] + [0.0022 × (Age _(years)_ × Leg length _(cm)_)] + [0.005841 × (Age _(years)_ × Trunk Height _(cm)_)] − [0.002658 × (Age _(years)_ × Weight _(kg)_)] + [0.07693 × (Weight _(kg)_/Height _(cm)_) × 100] (3)

(cm): Centimeters. (kg): Kilograms.

#### 2.2.8. Body Composition

The body composition, evaluated to characterize the sample, was measured by the indirect method of dual-energy X-ray absorptiometry–DXA (Prodigy Advance model–GE Lunar software, Madison - Wisconsin, USA). The evaluation allowed the identification of the following body components: Body mass index (BMI), percentage of total fat (% TF), bone mineral density (BMD), and fat-free mass (FFM) [23].

It is noteworthy that the DXA was configured with subroutines of appropriate algorithms for the pediatric population. Subsequently, the volunteers were placed in the supine position, on the surface of the device (dimension approximately 190 × 60 cm), which, by sliding the imaging device, at a distance of 80 cm and in a rectilinear manner, performed the scanning from the head to the feet, then, the detector captured the information associated with the attenuation of the photon beams and sent it for analysis on a microcomputer using specific software.

#### 2.2.9. Interventions: Guidelines for Healthy Eating

Nutritional guidelines were carried out in the three phases of the research, always at the end of data collection, in which the volunteer and their guardian received guidance from a researcher in the area of Nutrition, however, only the second and third assessments correspond to the results of nutritional counseling. The objective was to emphasize the importance of adequate food consumption for good development and to prevent or delay possible metabolic changes associated with the virus and the use of HAART. The guidelines were carried out based on the Food Guide for the Brazilian Population [24], in which foods are grouped according to the degree of processing.

Increased consumption of fresh and minimally processed foods, such as meats, tubers, fruits, vegetables, and cereals, was encouraged, and the consumption of ultra-processed foods, such as sausages, packaged snacks, fried snacks, and sweets, was discouraged. In the first stage, in addition to verbal guidance, those responsible for children and adolescents received the recommendations in print so that the guidelines could be consulted whenever necessary. This contained guidelines on healthy eating, cleaning fruits and vegetables, and a qualitative menu to serve as an example. Those responsible for the children needed to understand that a healthy diet based on fresh and minimally processed foods could be tasty, easy to obtain, and affordable.

In the second and third stages, as well as in the first, nutritional guidelines were given at the end of data collection. In both, the researcher in the area of nutrition talked to the participants and guardians, emphasizing the guidelines given in the first stage and clarifying the doubts and difficulties brought by them. During the research period, those responsible could get in touch via telephone message or call to clarify possible doubts.

#### 2.2.10. Guidance on Physical Activity

Throughout the evaluations, it was emphasized that the population did not decrease their healthy habits or did not present sedentary behaviors in different areas (at school, leisure, and free time). For example, when identified that they did not practice physical education at school, it was highlighted how negative it would be for this population’s motor, affective, and social development.

It was suggested the practice of physical exercises in other spaces (e.g., clubs and gyms) and participation in different sports outside the school environment (ballet, dance, swimming, football, futsal, among others), according to preferences and financial capacities. Finally, there was guidance to have more active free time habits, such as avoiding sitting for a long-time watching TV or playing on your cell phone or computer. Participants were also instructed to show active behavior throughout the day, such as buying groceries on foot or by bicycle and creating the habit of getting up to pick up objects. The orientation took place verbally, using examples to be followed.

**Figure 2:**
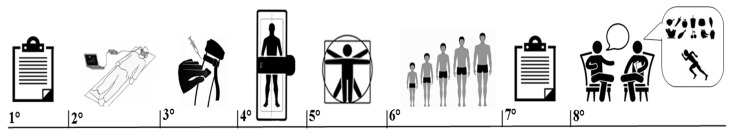
Order of assessments and guidelines for healthy habits. **1°**—Anamnesis. **2°**—Resting metabolic rate. **3°**—Venipuncture. **4°**—Analysis of body composition by dual-energy X-ray absorptiometry. **5°**—Anthropometric analyses. **6°**—Analysis of biological maturation. **7°**—Application of the questionnaires of habitual food consumption and level of habitual physical activity. **8°**—Guidance on healthy eating habits and the benefits of increasing the level of habitual physical activity.

Figure 2 shows the sequence of assessments and guidelines for healthy habits.

#### 2.2.11. Statistical Analysis

Only the data of the subjects who were in all three study evaluation periods were considered for the performance of the statistical tests. The normality of the data was verified by the Anderson-Darling and Z-score tests for asymmetry and kurtosis (−1.96 to 1.96). For the set of variables referring to the frequency of food intake, the assumption of normality was denied. Subsequently, the data were transformed (log base 10) from non-parametric to parametric. After the transformation of the data, comparative analyses were performed, and, for better visualization, the data were exposed in mean and standard deviation. Bonferroni’s correction was used in all comparative analyses. Analyses of covariance (ANCOVA) were carried out in the comparisons between the different evaluation periods of the present study [25]. In this sense, during ANCOVA, to control the variables sex, chronological age, and maturation, the Backdoor technique was used. Bonferroni’s post hoc test was used to indicate specific differences. The interaction effect of the periods on the analyzed variables was verified by η^2^p and the magnitude adopted was: Small η^2^p ≤ 0.10 to 0.23; mean η^2^p from 0.24 to 0.34; large η^2^p from 0.35 to 0.44; very large η^2^p ≥ 0.45 [26]. All analyses were performed using open-source software R (version 4.0.1; R Foundation for Statistical Computing^®^, Vienna, Austria), considering *p* < 0.05.

## 3. Results

Table 1 shows the socioeconomic characteristics of CAH, indicating a predominance of the group in elementary school, low family income, most with CD4 levels above 350 cells/mm^3^, ART time ranging from 1 to 15 years, and all under therapy.

There was a significant increase in body weight in analysis step 3 in relation to analysis step 1 (F (3.0) = 0.09, η^2^p = 0.004, *p* = 0.03). There was no significant variation in the other characteristics of the sample concerning the analysis periods performed by the present study (F (3.0) < 0.3, *p* > 0.06). However, there is a behavior of advancing skeletal and somatic maturation status during the periods of analysis, as well as maintenance of HPAL. RMR, on the other hand, decreased in periods 2 and 3 in relation to analysis period 1 (Effect of time: F (2.0) = 3.26, η^2^p = 0.20, *p* = 0.04). The variables chronological age, sex, and maturation did not present significant interactions during the comparative analysis (F (2.0) < 0.3, *p* < 0.05) (See Table 2).

Regarding the longitudinal effect of eating habits and level of physical activity on the biochemical parameters in CAH, a significant effect of time was identified concerning the analyzed variables (F (2.0) = 60.4, η^2^p = 0.976, *p* = 0.004). In addition, glucose levels decreased significantly in analysis periods 2 and 3 concerning analysis period 1. Triglyceride levels were lower in analysis 3 concerning analysis 1 (See Table 3).

There was an increase in HDL cholesterol and urea in the period of analysis 3 concerning periods 1 and 2. Creatinine was higher in the period of analysis 2 concerning period 3. The variables’ chronological age, sex, and maturation did not result in significant interactions during comparative analysis (F (2.0) < 0.06, *p* < 0.05).

Concerning 24HR, it was observed that in all phases, there was low adequacy of the total energy consumed by the participants, estimated and evaluated individually according to weight and age. It was also seen that most of the time, the participants were not able to consume the micronutrient values stipulated by the DRI (17). However, there was no significant effect of time (F (2.0) = 1.11, η^2^p = 0.15, *p* = 0.3) (See Table 4). In addition, there was no significant difference between the 24HR variables in the analysis periods. There were no significant interactions during the comparative analyses (F (2.0) < 0.7, *p* < 0.05) in the variables chronological age, sex, and maturation.

Regarding eating habits, there was no significant effect of time (F (2.0) = 2.61, η^2^p = 0.23, *p* = 0.1). However, there was a significant increase in fruit consumption in analysis 3 concerning periods 1 and 2 (See Table 5). The consumption of soft drinks decreased in the period of analysis 2 and increased again in analysis 3. For the variables chronological age, sex, and maturation, there were no significant interactions during the comparative analyses (F (2.0) < 1.02, *p* < 0.05).

## 4. Discussion

The present study aimed to analyze the influence of dietary counseling and physical activity on biochemical and metabolic parameters in children and adolescents with HIV. It was found that counseling positively influenced healthy habits, and these, in turn, improved health-related parameters. There was a significant effect of time on the reduction of blood glucose and triglycerides and metabolic rest rate, despite being within the normal range since the first assessment (analysis 1). There were no significant changes in the level of physical activity, as well as in the quantitative parameters of food consumption over time. However, there was a significant increase in fruit consumption, a reduction in the consumption of soft drinks in the period of analyses 1 and 2, with an increase in the period of analysis 3.

Regarding the effect of the intervention on the biochemical parameters in CAH, in addition to changes in glycemia and triglycerides, we also observed a significant reduction in LDL between analyses 1 and 2 and an increase in analysis 3. The maintenance of blood glucose, triglycerides, and LDL levels, within the reference values for the pediatric population, may be an indicator of the improvement of some markers of food consumption, such as the increase in fruit consumption and a reduction in the frequency of consumption of soft drinks [27]. There was a momentary reduction in LDL, as well as in the behavior of the frequency of consumption of soft drinks, which reinforces the need for nutritional guidelines even more frequently. In this context, it is evident that food education needs to happen continuously, as HIV-infected children undergoing treatment tend to be at high risk for dyslipidemia, especially high triglycerides and low HDL [28,29].

About the effect of time on the reduction of the resting metabolic rate, it is noteworthy that the CAH, since the first analysis, showed an adequate behavior, being maintained over time, which corroborates with a study, although cross-sectional, which identified that perinatally infected children are not hypermetabolic and maintain normal growth when they provide adequate nutritional status [30].

It was observed in the quantitative food consumption of CAH over time, the inadequacy of the predominant energy consumption in the three phases, in which most of the participants with inadequate intake did not reach the estimated TEV, corroborating with other studies that also found inadequacies in food consumption, however, with consumption above the estimated TEV [31,32]. As for the distribution of macronutrients in food consumption, consumption consistent with the recommendations proposed by DRIs [18] was observed, except fat consumption, in the second phase, in which it was below that recommended by the guideline.

In all phases of the study, low fiber consumption was observed, especially in the second and third phases, in which 100% of the participants had a consumption below 25 g/day, according to DRI [18], results similar to those of Shiau et al. [31], in which 95.9% of the population had fiber consumption below that proposed by the DRIs, in addition to the average consumption of 12.4 g/day. In contrast, Hillesheim et al. [32] obtained as results the average consumption and the percentage of participants above that recommended by the DRI [18].

The low intake of micronutrients observed in the present study may be associated with higher consumption of processed foods (ultra-processed). Although there are no significant differences, it was observed in all stages of the research, low consumption of micronutrients, naturally present in fresh and minimally processed foods [24]. Such behavior can harm CAH, as micronutrients play important roles in the synthesis of hormones and proteins, maintenance of the immune system, in addition to the antioxidant role [33], capable of minimizing the inflammatory action of the virus and the chronic consumption of antiretroviral therapy [34].

Although there was no difference in the consumption of dietary fiber, we found a significant increase in the frequency of fruit consumption, pointing to positive behavior that would possibly impact in the long run if such behavior were maintained. We emphasize that the orientation towards fruit consumption was based on local (from the region) fruit options, which are easy to purchase and affordable, a strategy suggested in a review study [1].

Regarding the consumption of soft drinks, there was a reduction in analysis period 2, but in contrast, there was a greater consumption in analysis period 3. On this result, it is known that nutritional orientation is fundamental, however, the socio-economic context and cultural aspects that directly reflect on eating habits or behaviors of people with HIV can hinder changes or lifestyle habits, especially in children and adolescents. This result, associated with other factors, reinforces the need for adults (parents or guardians) and CAH to be monitored and guided by health professionals [30].

Furthermore, even though there is no significant difference, it is highlighted that counseling on the level of habitual physical activity is important for the maintenance and improvement of the general health of CAH, as the group did not reduce the level of physical activity over time, usually perceived from different forms of sedentary behavior in young people with HIV, such as participation in sedentary games from personal access to devices such as cell phones, internet services, and televisions, which makes them spend long hours sitting [35,36]. It is noteworthy that the level of habitual physical activity should be encouraged and explained about its importance for family members, since interpersonal factors may also be related to the sedentary lifestyle of the pediatric population with HIV, as parenting practices that tolerate physical inactivity are evidenced, such as confining the child inside the house or not giving permission to play sports [36].

It is known that nutritional counseling and exercise interventions in adults with HIV are effective in treating obesity, fat redistribution, and metabolic abnormalities, however, in the pediatric population, investigations need to be further examined [1,2,3,4,5,6,7,8,9,10,11,12,13,14,15,16,17,18,19,20,21,22,23,24,25,26,27,28,29,30,31,32,33,34,35,36,37]. Evidence suggests that an approach of anticipation and accountability for nutrition and concern with the level of physical activity can improve the results and help minimize the adverse consequences (metabolic, cardiovascular, and psychological) of HIV [1,38,39], mainly during the development and maturation phases [3].

Among the challenges or limitations of the present study, the absence of a control group composed of children without HIV, the difficulty encountered in recruiting volunteers and remaining in the longitudinal study stands out, as well as effectively participating in a robust intervention, as the parents mentioned not having time or availability, difficulties in locomotion due to at a distance and not having the financial means to move with their children to participate in an intervention with physical exercises. Therefore, it is suggested to consider the socioeconomic profile of this population, motivate the children’s parents, and to establish accessible strategies so that this public can be fully assisted to health, as the importance of establishing ways that provide an adequate standard of care for CAH [2] is already evident.

## 5. Conclusions

Counseling healthy habits and regular clinical follow-up were relevant for improving biochemical parameters (glucose, triglyceride, HDL cholesterol), maintaining the resting metabolic rate, increasing fruit consumption, and decreasing the consumption of soft drinks, in part of the time, of children and adolescents with HIV. However, it did not promote significant changes in the level of habitual physical activity and food consumption over time. Finally, we emphasize that counseling related to food and physical activity was able to influence healthy habits, which promoted improvements in health-related parameters. It is suggested to carry out multidisciplinary work, with meetings held more frequently, in addition to carrying out dynamic activities, which generate the interest of children and adolescents with HIV in promoting the development of healthy habits, and, consequently, minimize the impacts of infection in this population. In addition, it is necessary to motivate the children’s parents, as difficulties were observed in implementing actions or projects with more robust interventions.

## Figures and Tables

**Table 1:** HIV + group characteristics (*n* = 18).

Subject No.	Educational Stage	Per Capita Income (Brazilian Currency)	CD4 (Cells/mm^3^)	ART (Years)	Therapy
C1	First grade	360.00	855	6	AZT/3TC/NVP
C3	First grade	159.00	451	2	AZT/3TC/EFZ
C4	Fifth grade	238.50	1.038	11	3TC/AZT/LPV/r
C5	Preschool	181.30	1.709	5	AZT
C6	First grade	238.50	2.372	6	LPV/r/AZT/3TC
C7	Tenth grade	1192.50	147	1	TDF/3TC/LPV
C8	Sixth grade	381.60	955	14	3TC/TDF/LPV
C9	Ninth grade	381.60	1.326	17	3TC/ITRN/IP/II
C10	Tenth grade	545.10	585	15	TDF/3TC/LPV
C11	First grade	190.80	282	0	AZT/LMP/EFZ
C12	Second grade	572.40	366	1	AZT/3TC/EFZ
C13	Fifth grade	715.50	799	6	3TC/AZT/NVP
C14	Seventh grade	175.00	1.060	9	AZT/3TC/EFZ
C15	Fourth grade	190.80	NI	11	AZT/3TC/LPV
C16	Second grade	106.00	3.365	3	AZT/3TC/EFZ
C17	Third grade	159.00	507	1	AZT/3TC/EFZ
C18	Sixth grade	477.00	545	14	TDF/3TC/LPV
C19	Third grade	17.50	767	2	AZT/3TC/EFZ

CD4: CD4 lymphocytes; ART: Antiretroviral therapy; NI: Noninformed; 3TC: Lamivudine; LPV/r: Lopinavir + ritonavir; EFZ: Efavirenz; AZT: Zidovudine; NVP: Nevirapine; IP: Ritonavir; ITRN: Tenofavir; IP: Darunavir; II: Rautegravir. Data presented in a descriptive way.

**Table 2:** The behavior of the variables body composition, bone mineral density, habitual physical activity level, and resting metabolic rate of the sample in each period of analysis.

Variables	Analyses		
	1	2	3
Height (m)	1.40 ± 0.20	1.42 ± 0.22	1.47 ± 0.10
Body weight (kg)	32.2 ± 12.4	34.0 ± 14.0	* 39.6 ± 9.57
Body mass index (kg/m^2^)	15.6 ± 2.10	15.6 ± 2.33	18.1 ± 2.75
Total F (%)	24.5 ± 6.13	22.2 ± 5.06	21.4 ± 9.92
Fat free mass (kg)	21.2 ± 9.44	20.0 ± 12.8	20.0 ± 11.4
Bone mineral density (g/cm2)	0.78 ± 0.18	0.71 ± 0.33	0.67 ± 0.28
Physical activity index at school	2.30 ± 0.57	2.47 ± 0.55	2.48 ± 0.45
Physical activity index active leisure	2.42 ± 1.31	2.45 ± 1.07	2.31 ± 1.01
Index of physical activity in free time	2.52 ± 0.53	1.57 ± 0.83	2.57 ± 0.67
Habitual physical activity index	7.23 ± 2.04	7.46 ± 2.05	7.35 ± 1.63
Resting metabolic rate (Kcal/day)	0.79 ± 0.20	* 0.57 ± 0.45	* 0.62 ± 0.42

Total F%: Fat percentage; 1: Evaluation at the beginning; 2: Evaluation in the 4th month; and 3: Evaluation in the 8th month; * Statistical difference only concerning analysis 1.

**Table 3:** Longitudinal effect of eating habits and guidelines for improving physical activity on biochemical parameters in children and adolescents with HIV.

Variables	Analyses			F_(2.0)_	η^2^p	*p*
	1	2	3			
Glucose (mg/dL)	§ 76.8 ± 36.4	64.5 ± 42.1	62.7 ± 33.9	0.16	0.50	0.007
Triglycerides (mg/dL)	Ϟ 85.4 ± 61.3	44.7 ± 49.5	56.6 ± 41.9	4.53	0.60	0.01
Total cholesterol (mg/dL)	116.0 ± 67.6	96.4 ± 79.9	111.4 ± 64.6	1.84	0.38	0.2
HDL-C (mg/dL)	27.4 ± 23.5	26.0 ± 21.0	31.4 ± 19.6	0.29	0.08	0.62
LDL-C (mg/dL)	55.4 ± 45.8	47.9 ± 54.5	# 68.7 ± 46.8	10.7	0.78	0.04
Urea (mg/dL)	11.2 ± 7.60	12.2 ± 11.1	# 17.3 ± 9.84	11.7	0.79	0.04
Creatinine (mg/dL)	0.50 ± 0.34	ư 0.56 ± 0.28	0.43 ± 0.27	36.8	0.92	0.01

1: Evaluation at the beginning; 2: Evaluation in the 4th month; and 3: Evaluation in the 8th month; LDL-C: Low-density lipoprotein cholesterol; HDL-C: High-density lipoprotein cholesterol. § Statistical difference concerning analyses 2 and 3. Ϟ Difference only in relation to analysis 3. # Statistical difference concerning analyses 1 and 2. ư Statistical difference concerning analysis 3.

**Table 4:** Analysis of 24HR over time of children and adolescents with HIV.

Variables	Analyses			F_(2.0)_	η^2^p	*p*
	1	2	3			
ETEV: (Kcal)	1942.6 ± 459.9	1570.7 ± 928.0	1977.1 ± 548.7	1.67	0.12	0.2
KCAL CONSUMED	1900.5 ± 507.5	1449.5 ± 1013.0	17995 ± 503.9	1.00	0.10	0.1
Did not reach ETEV:	94.5%	77.8%	90%	-----	-----	-----
>ETEV:	27.8%	33.3%	30%	-----	-----	-----
<ETEV:	66.7%	44.5%	60%	-----	-----	-----
Proteins	70.4 ± 19.3	54.8 ± 39.8	79.0 ± 24.0	1.38	0.10	0.4
%_PROT	14.5 ± 4.27	12.5 ± 6.68	17.8 ± 3.29	1.91	0.13	0.1
Lipids	56.9 ± 16.0	52.5 ± 43.1	51.9 ± 21.3	4.21	0.26	0.06
%_LIP	27.2 ± 3.41	21.8 ± 15.6	26.2 ± 5.86	2.11	0.15	0.1
Carbohydrates	271.4 ± 84.5	178.6 ± 127.3	252.3 ± 77.3	0.84	0.06	0.3
%_CARB	57.1 ± 4.28	44.0 ± 23.4	56.0 ± 5.91	1.00	0.07	0.33
Total cholesterol (mg)	251.1 ± 114.3	241.6 ± 224.7	247.6 ± 94.4	0.07	0.00	0.7
Fibers (g)	18.6 ± 7.01	12.2 ± 7.36	15.1 ± 6.18	0.01	0.00	0.9
<DRI	88.9%	100%	100%	-----	-----	-----
Calcium (mg)	480.4 ± 258.8	386.3 ± 320.3	382.8 ± 297.5	0.02	0.00	0.8
<DRI	94.45%	88.9%	100%	-----	-----	-----
Magnesium (mg)	156.5 ± 59.4	118.6 ± 75.4	173.9 ± 47.8	0.00	0.00	0.9
<DRI	83.33%	77.79%	50%	-----	-----	-----
Phosphorus (mg)	1508.6 ± 2134.6	784.6 ± 586.1	1002.3 ± 495.9	0.36	0.02	0.5
<DRI	61.10%	44.45%	40%	-----	-----	-----
Iron (mg)	7.11 ± 3.25	5.90 ± 3.94	9.18 ± 3.86	2.59	0.17	0.1
<DRI	100%	88.89%	60%	-----	-----	-----
Potassium (mg)	1675.7 ± 394.3	1272.9 ± 842.4	1807.1 ± 539.3	2.13	0.15	0.16
<DRI	100%	100%	100%	-----	-----	-----
Zinc (mg)	7.14 ± 3.61	6.32 ± 4.59	10.1 ± 5.35	0.11	0.00	0.7
<DRI	61.11%	44.45%	20%	-----	-----	-----

1: Evaluation at the beginning; 2: Evaluation in the 4th month; and 3: Evaluation in the 8th month; ETEV: Estimated total energy value; KCAL: Kilocalorie; PROT: Proteins; LIP: Lipids; CARB: Carbohydrates; DRI: Dietary reference intakes.

**Table 5:** Analysis of frequency and food consumption of children and adolescents with HIV over time.

Variables	Analyses			F_(2.0)_	η^2^p	*p*
	1	2	3			
Raw Salad	0.56 ± 0.78	0.73 ± 1.27	1.00 ± 2.00	1.06	0.05	0.3
Vegetables	1.50 ± 1.75	1.73 ± 2.76	1.40 ± 2.22	0.01	0.00	0.9
Fruit	2.94 ± 2.85	2.45 ± 3.04	# 3.70 ± 2.86	5.04	0.22	0.03
Bean	4.67 ± 2.74	3.09 ± 3.04	4.10 ± 3.54	0.00	0.00	0.9
Milk and dairy products	3.94 ± 3.09	2.64 ± 3.17	4.10 ± 2.68	0.07	0.00	0.7
Frying	1.61 ± 2.47	0.64 ± 1.20	1.40 ± 2.06	0.00	0.00	0.9
Embedded Foods	2.33 ± 1.97	1.64 ± 2.15	1.70 ± 1.63	0.10	0.00	0.7
Salty	2.56 ± 2.87	1.27 ± 1.48	2.20 ± 2.34	1.95	0.10	0.1
Candy	2.28 ± 2.49	1.18 ± 2.13	2.10 ± 2.80	0.00	0.00	0.9
Soft drinks	° 1.44 ± 1.38	0.55 ± 0.82	° 1.43 ± 1.50	5.72	0.25	0.02

1: Evaluation at the beginning; 2: Evaluation in the 4th month; and 3: Evaluation in the 8th month; ° Statistical difference concerning analysis 2. # Statistical difference concerning analyses 1 and 2.

## Data Availability

The data that support the findings of this study are available from the corresponding author upon reasonable request.
